# Supplementary material for: Spatial Distribution of Soil Heavy Metals and Associated Environmental Risks near Major Roads in Southern Tibet, China
Source: Int J Environ Res Public Health. 2022 Jul 8;19(14):8380. doi: 10.3390/ijerph19148380 (PMC9318444; doi:10.3390/ijerph19148380)
Supplement: Supplementary file 1 [file ijerph-19-08380-s001.zip › ijerph-1754232-supplementary.pdf]

Table S1: Information on the traffic of the major roads in the southern part of the Tibetan Plateau

**Road number**	**The flow of passenger vehicles**	**speed(km/h)**	**The flow of truck**	**speed(km/h)**	**The flow of car**	**speed(km/h)**	**The flow of motor vehicles**	**speed(km/h)**	**Mixed flow**	**Congestion ratio**	**Number of trips （pcu·km/d）**	**Number of dates**	**Number of hours**	**Real-time vehicles**
G318	1523	47.87	812	41.33	2334	45.65	2485	45.27	2485	0.35	110570	265	6312	136926
G562	890	44.50	527	41.03	1416	42.73	1619	40.83	1619	0.39	70200	231	5502	120390
G219	755	57.60	323	49.34	1073	54.49	1139	53.74	1139	0.26	43717	296	6941	141595
G216	339	20.95	214	15.85	553	19.50	622	18.90	622	0.27	49018	234	5597	98772
